# Stationary Phase-Specific Virulence Factor Overproduction by a *lasR* Mutant of *Pseudomonas aeruginosa*

**DOI:** 10.1371/journal.pone.0088743

**Published:** 2014-02-12

**Authors:** Matthew T. Cabeen

**Affiliations:** Department of Molecular and Cellular Biology, Harvard University, Cambridge, Massachusetts, United States of America; Ghent University, Belgium

## Abstract

Secreted virulence factors of the human pathogen *Pseudomonas aeruginosa* are often under quorum sensing control. Cells lacking the quorum-sensing regulator LasR show reduced virulence factor production under typical laboratory conditions and are hypo-virulent in short-term animal infection models, yet *lasR* mutants are frequently associated with long-term infection in cystic fibrosis patients. Here, I show that in stationary-phase or slow-growth conditions, *lasR* cells continuously and strongly produce the important virulence factor pyocyanin while wild-type cells do not. Pyocyanin overproduction by *lasR* cells is permitted by loss of repression by RsaL, a LasR-dependent negative regulator. *lasR* cells also contribute pyocyanin in mixed cultures, even under “cheating” conditions where they depend on their wild-type neighbors for nutrients. Finally, some clinical *P. aeruginosa* isolates with *lasR* mutations can overproduce pyocyanin in the laboratory. These results imply that slow-growing clinical populations of *lasR* cells in chronic infections may contribute to virulence by producing pyocyanin under conditions where *lasR* ^+^ cells do not.

## Introduction

**Figure 1:**
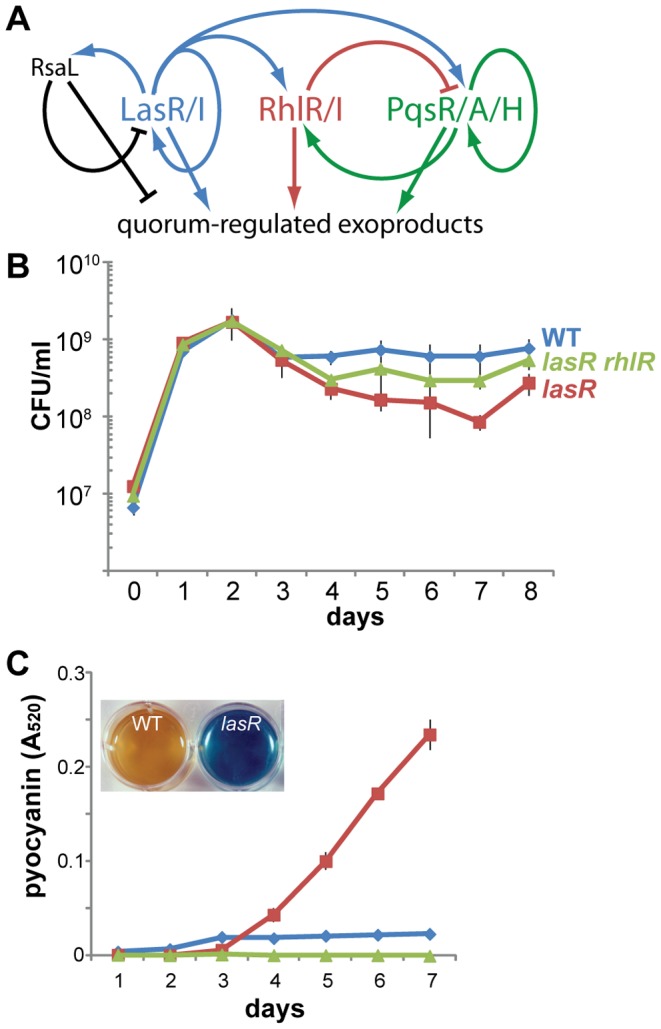
A *lasR* mutant overproduces pyocyanin under stationary-phase culture conditions. A. A simplified diagram showing some of the regulatory pathways linking the three quorum-sensing systems of *P. aeruginosa* and exoproduct synthesis. Each system and its regulatory pathways is color coded. B. Plot of the number of live cells (colony-forming units) in static cultures of strains with wild-type (WT) PA14 (MTC772), PA14 *lasR* (MTC774), and PA14 *lasR rhlR* (MTC797) backgrounds in LB at 25°C. C. Pyocyanin quantification in static cultures of wild-type (WT) PA14 (MTC1), PA14 *lasR* (MTC390), and PA14 *lasR rhlR* (MTC626) in LB at 25°C. The inset image shows the appearance of the cultures in a 12-well plate after 7 d. All plots show the mean ± standard deviation of 3 biological replicates.

*Pseudomonas aeruginosa* is a common opportunistic bacterial pathogen that causes human infections in a variety of clinical situations [1] but is especially important in cystic fibrosis lung infections [2]. Many of the numerous virulence factors produced by *P. aeruginosa* are under the control of quorum sensing, which uses diffusible autoinducer molecules as a way to monitor cell density [3]. Specific genes are thus activated when bacterial cell population density, and hence autoinducer concentration, exceeds a threshold. *P. aeruginosa* has at least three quorum-sensing systems, with distinct autoinducer molecules and partially overlapping regulons, that are hierarchically arranged. The Las system is the first to become activated, and it in turn stimulates additional systems known as Rhl and PQS, which additionally regulate each other (Fig. 1) [3]. Finally, pyocyanin, a phenazine small molecule and virulence factor, acts as a terminal signaling factor in the quorum-sensing cascade [4]. Consistent with this hierarchy, inactivation of LasR, the regulatory protein of the Las quorum-sensing system, has been reported to severely attenuate quorum sensing, the production of quorum-regulated factors, and virulence in typical laboratory culture and in short-term animal models [5], [6], [7], [8], [9], [10], [11].

Decreased quorum sensing can permit *lasR* mutants to become social cheaters that gain a growth advantage by utilizing quorum-regulated “public goods” (including virulence factors) produced by nearby wild-type cells rather than producing their own [12], [13]. Cheating was thus proposed as one reason [13] why *lasR* mutants are detected in highly chronic human infections such as those occurring in cystic fibrosis patients [14]. Consistent with this idea, *lasR* mutant cells outcompeted co-infected wild-type cells and lowered overall virulence in a murine burn-infection model, consistent with their being non-producing cheaters [11]. However, recent work has revealed that cells lacking LasR function can in fact accomplish quorum sensing by employing the Rhl and PQS systems [15], [16]. Without activation by LasR, the quorum response is substantially delayed, but it appears to resemble the wild-type response in terms of gene expression and virulence factor synthesis [15]. Such LasR-independent virulence factor production is another potential explanation for why *lasR* mutants may not reduce overall virulence in long-term cystic fibrosis infections. In accord with this idea, the presence of *lasR* mutant cells has been correlated with disease progression and declining lung function in cystic fibrosis patients [17].

Pyocyanin is one of the most important quorum-regulated virulence factors of *P. aeruginosa*. It has numerous toxic effects on host tissues at such infection sites as the respiratory epithelium, where its toxicity is thought to be related to the generation of reactive oxygen species when pyocyanin is oxidized [18]. Pyocyanin is under the control of the Rhl and PQS systems and can accordingly be produced even in the absence of LasR after a delay [15]. As with the presence of *lasR* mutants, high levels of sputum pyocyanin have been associated with advanced infection in cystic fibrosis patients [19]. Pyocyanin also serves as an antibiotic thanks to its redox activity, can act as a terminal electron acceptor for *P. aeruginosa*, and is a terminal signaling molecule in the quorum-sensing cascade [4]. It is therefore useful for monitoring quorum-sensing activity in *P. aeruginosa*, especially given its bright blue color when oxidized.

Most previous laboratory studies of *P. aeruginosa* quorum sensing have observed bacteria exponentially growing in shaking culture. Under such conditions, wild-type quorum-sensing behaviors begin during late exponential phase and continue into stationary phase, while LasR-independent behaviors do not begin to appear until the onset of stationary phase, between 8 and 24 h [15]. However, most bacterial cells in nature are not growing in optimal laboratory-like conditions. Instead, pathogens often form biofilms during infection [20]. The physiology of slowly growing bacteria resembles that of bacteria growing in biofilms [21], and *P. aeruginosa* expresses the stationary-phase sigma factor RpoS both in clinical sputum samples [22] and in continuously fed biofilms in vitro [23]. Indeed, one reason for the treatment resistance of cells growing in biofilms is their relatively slow growth [24]. Therefore, I reasoned that slow-growing or stationary-phase cells maintained in longer-term culture might manifest phenotypes that reflect their behavior in a more physiologically relevant state. Here, I report that wild-type and *lasR* cells exhibit clearly distinct yet complementary stationary-phase phenotypes. Moreover, wild-type/*lasR* mixtures can collaborate to enact behaviors inaccessible to the individual strains.

## Materials and Methods

### Routine bacterial culture

*Pseudomonas aeruginosa* and *Escherichia coli* strains were routinely cultured on LB Lennox solid (1.5% agar) and liquid media at 37°C. Culture stocks were stored in 25% glycerol at -80°C, and fresh plates were grown for each experiment. The following antibiotics were used for selection/maintenance for *P. aeruginosa*; the maintenance concentration was used for *E. coli* culture: gentamycin (75/15 µg/ml) and tetracycline (75/25 µg/ml). Irgasan (25 µg/ml) was used as an *E. coli*-specific selective agent. *P. aeruginosa* strains are listed in Table 1. *E. coli* strains are listed in Table S1 in File S1, plasmids are listed in Table S2 in File S1, and primer sequences are listed in Table S3 in File S1.

**Table 1:** Strains used in this study.

Strain	Alias	Relevant genotype or description	Source or reference
*P. aeruginosa*			
MTC1		PA14	
MTC63		PA14 Δ*phz*	[4]
MTC390		PA14 Δ*lasR*	This study
MTC498		PA14 Δ*rsaL*	This study
MTC500		PA14 Δ*rsaL* Δ*lasI*	This study
MTC537		PA14 Δ*pqsA*	This study
MTC556		PA14 Δ*rhlI* Δ*pqsA*	This study
MTC625		PA14 Δ*lasR* Δ*rhlI*	This study
MTC626		PA14 Δ*lasR* Δ*rhlR*	This study
MTC628		PA14 Δ*lasR* Δ*pqsA*	This study
MTC637		PA14 Δ*lasR attB*::*CTX-1-aacC1* (Gent^R^)	This study
MTC723		PA14 *attB*::*CTX-1-P_lasB_-lux*	This study
MTC725		PA14 *attB*::*CTX-1-P_phzA1_-lux*	This study
MTC733		PA14 *attB*::*CTX-1-P_hcnA_-lux*	This study
MTC735		PA14 *attB*::*CTX-1-P_rhlA_-lux*	This study
MTC737		PA14 *attB*::*CTX-1-P_rsaL_-lux*	This study
MTC745		PA14 Δ*lasR attB*::*CTX-1-P_lasB_-lux*	This study
MTC747		PA14 Δ*lasR attB*::*CTX-1-P_phzA1_-lux*	This study
MTC755		PA14 Δ*lasR attB*::*CTX-1-P_hcnA_-lux*	This study
MTC757		PA14 Δ*lasR attB*::*CTX-1-P_rhlA_-lux*	This study
MTC759		PA14 Δ*lasR attB*::*CTX-1-P_rsaL_-lux*	This study
MTC772		PA14 *attB*::*CTX-1-lux*	This study
MTC774		PA14 Δ*lasR attB*::*CTX-1-lux*	This study
MTC789		PA14 Δ*lasR* Δ*rhlR attB*::*CTX-1-P_lasB_-lux*	This study
MTC790		PA14 Δ*lasR* Δ*rhlR attB*::*CTX-1-P_phzA1_-lux*	This study
MTC794		PA14 Δ*lasR* Δ*rhlR attB*::*CTX-1-P_hcnA_-lux*	This study
MTC795		PA14 Δ*lasR* Δ*rhlR attB*::*CTX-1-P_rhlA_-lux*	This study
MTC797		PA14 Δ*lasR* Δ*rhlR attB*::*CTX-1-lux*	This study
MTC838		PA14 Δ*lasR* Δ*ambB*	This study
MTC842		PA14 Δ*lasR* Δ*phoB*	This study
MTC949		PA14 Δ*lasR* Δ*rhlI* Δ*pqsA*	This study
0007-2	CF1	*lasR* wild type (confirmed by sequencing)	[17]
0022-1	CF2	*lasR*_179C>T_ (W60STOP identified by sequencing)	[17]
0024-5	CF3	*lasR* wild-type (identified by sequencing)	[17]
0029-1	CF4	*lasR*_557T>G_ (Q186P identified by sequencing)	[17]
0053-2	CF5	*lasR*_520G>A_ (P174S identified by sequencing)	[17]
0063-6	CF6	*lasR*Δ_350–362_ (deletion and frameshift identified by sequencing)	[17]

### Specialized media

M63 medium contained 100 µM (13.6 g/L) KH_2_PO_4_, 15.14 mM (2 g/L) (NH_4_)_2_SO_4_, and 0.36 µM (0.1 mg/L) FeSO_4_·H_2_O. A 5X salts stock was adjusted to pH 7.0 with KOH before autoclaving. To make the final medium, the 5X stock was mixed with 0.2% casamino acids and 0.5% glycerol from 20% and 50% sterile stocks, respectively, and adjusted to 1X with sterile H_2_O.

M9 medium was based on a salt solution of 12.8 g/L NaHPO_4_·7H_2_O, 3 g/L KH_2_PO_4_, 0.5 g/L NaCl, 1 g/L NH_4_Cl. A 5X salts stock was prepared and autoclaved. To make the final medium, the 5X stock was mixed with 2 mM MgSO_4_ and 0.1 mM CaCl_2_ from sterile 1M stocks, the appropriate carbon sources (sodium caseinate was added from a 10% autoclaved stock), and was adjusted to 1X with sterile H_2_O.

SCFM medium was made as described by Palmer *et al.* [25] and was prepared and used freshly, as it displayed a short shelf life.

### Specialized culture conditions

Static cultures of *P. aeruginosa* were grown in 4-ml volumes in 12-well microtiter plates, in 2-ml volumes in 24-well plates, or in 200-µl volumes in 96-well plates. A 1% volume of stationary-phase LB starter culture (OD_600_∼2-3), adjusted to OD_600_ = 1.0, was used for inoculation (hence, the starting OD_600_ was equivalent to 0.01). Pure autoinducer molecules (3OC12-HSL, C4-HSL, and PQS; Sigma) were added from 100 mM stocks in DMSO, and equivalent volumes of DMSO were used for controls.

### Reporter assays

Luminescent *lux* reporter strains were grown in static 200-µL LB cultures in 96-well microtiter plates. The cultures were inoculated at an initial OD_600_ of 0.01 (from starter cultures diluted to OD_600_ = 1.0) and grown at 25°C and 40% relative humidity. At daily time points, the luminescence values of the plate were read with a BioTek Synergy 2 plate reader. Data were collected using BioTek Gen5 software. At the same time points, static cultures with isogenic backgrounds, bearing an empty reporter and prepared identically, were taken, vortexed to disperse cells (effective dispersion was verified by phase-contrast microscopy) and serially diluted to obtain colony-forming unit (CFU) counts. The resulting CFU counts were then used to normalize the luminescence values of their respective reporter strains.

### Pyocyanin extraction and quantification

Pyocyanin was extracted from the supernatants of liquid cultures by adding an equal volume of chloroform (CHCl_3_) and vigorously vortexing. The lower, pyocyanin-containing organic layer was then taken and vortexed with an equal volume of 0.2 M HCl. The pink pyocyanin-containing aqueous layer was taken, and its absorbance at 520 nm (A_520_) was read in a BioTek Synergy 2 plate reader.

For some experiments, pyocyanin was quantified directly in culture supernatants by reading the absorbance at 691 nm as previously described [19]. Sterile medium was used as a blank.

### Cheating experiments

For cheating experiments, PA14 cells or mixtures of PA14 or PA14 *phz* cells with *lasR* cells were grown in liquid M9 medium (6 mL) containing 1% casein at 25°C in a tube roller. Pyocyanin was quantified as described above. The fraction of *lasR* cells within a mixture was determined using a *lasR* strain chromosomally marked with gentamycin resistance (MTC637). Cultures were serially diluted in 1X M9 salts and plated on LB or LB containing 5 µg/ml gentamycin to obtain CFU counts.

### Statistical analysis

Comparisons between samples were analyzed using unpaired equal-variance two-tailed Student's t-tests. The threshold for significance was set as *p*<0.01.

## Results

### Pyocyanin is overproduced by *lasR* cells in extended stationary-phase culture

To observe the behavior of stationary-phase cells over a time period of days rather than hours, as in traditional laboratory studies, I examined static liquid LB cultures of PA14 and a *lasR* mutant derivative growing at 25°C. Under these conditions, cells grow to a high density that then very gradually falls over the course of several days (Fig. 1B) but do not exhibit the “death phase” that typically precedes long-term adaptation to stationary phase [26]. In shaking culture, wild-type cells produce noticeable pyocyanin beginning in late exponential phase [4], while *lasR* cells begin to produce it by 24 h of culture [15]. After 3–4 days in static culture, I unexpectedly observed strong and continuing production of pyocyanin by stationary-phase *lasR* cells that turned the cultures dark blue, while wild-type cells produced virtually no visible pyocyanin at any time during the experiment (Fig. 1C). This effect was strongest in LB at 25°C, but the same trend appeared in static cultures of minimal M63 medium and in a nutritional mimic of cystic fibrosis sputum [25] at both 25°C and 37°C (Fig. S1 in File S1). Therefore, the wild type and *lasR* mutant display distinct stationary-phase phenotypes in that *lasR* cells continually produce pyocyanin while wild-type cells barely produce any pyocyanin. The phenotype of the *lasR* mutant was not due to additional mutations accumulated during the experiment, as cells from 6-day-old blue cultures displayed the same time course of pyocyanin production when inoculated into liquid LB, grown overnight at 37°C, and re-inoculated into static LB (Fig. S2 in File S1).

### Stationary-phase wild-type and *lasR* cells express distinct quorum-regulated virulence genes

**Figure 2:**
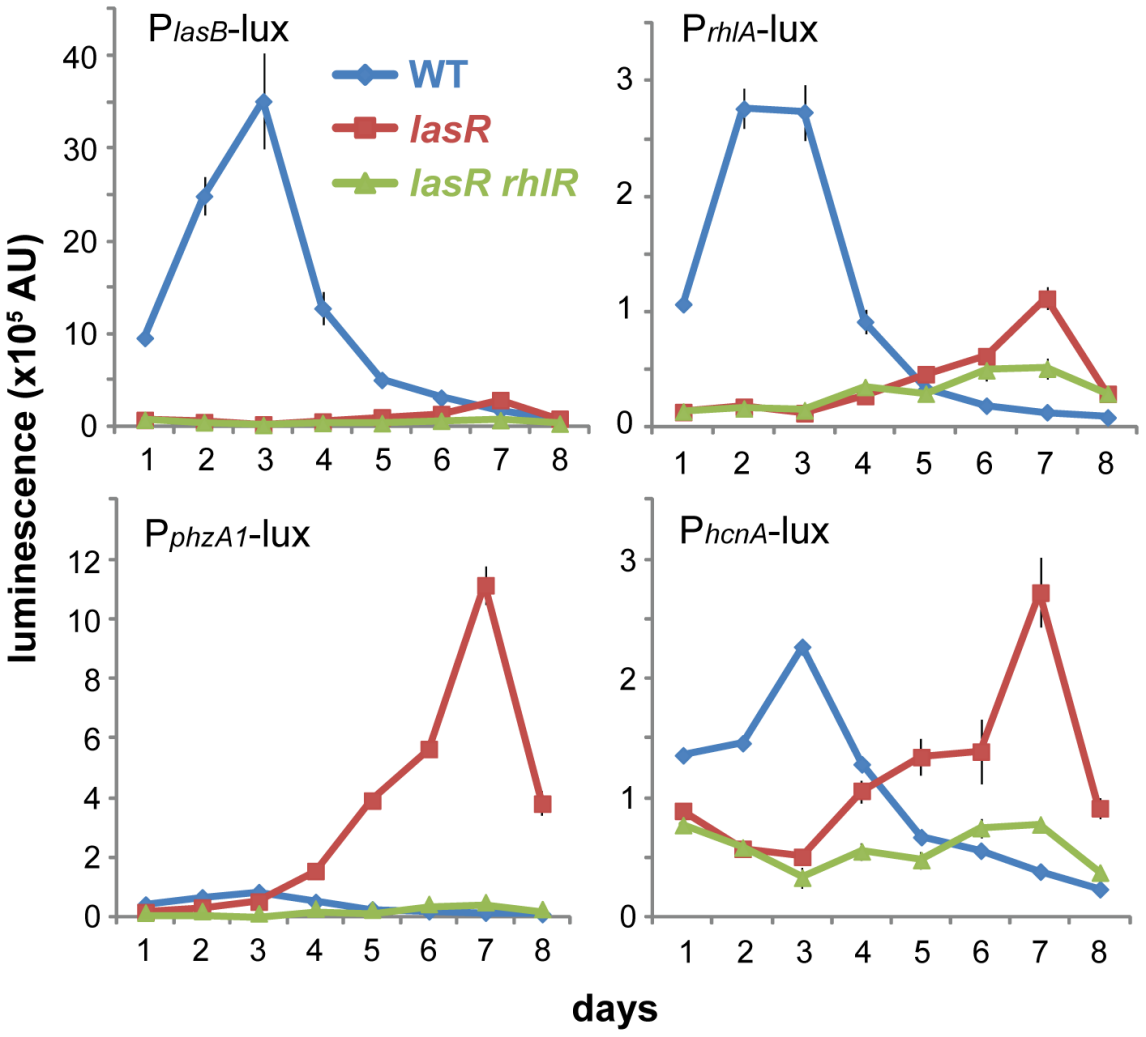
Wild-type and *lasR* mutant cells display distinct virulence gene expression patterns in stationary-phase culture. Gene expression was measured using chromosomally integrated *lux* reporters in the listed strain backgrounds. Plots show the mean ± standard deviation of 3 biological replicates each composed of 3 technical replicates.

Because stationary-phase wild-type and *lasR* cells displayed distinct phenotypes with respect to pyocyanin production, I analyzed the expression of additional quorum-regulated genes with roles in virulence factor production. Two distinct expression patterns were apparent. The first, typified most strongly by *lasB* but also seen for *rhlA* (encoding the LasB elastase and a rhamnolipid biosynthesis protein, respectively), showed strong early expression in the wild-type but only weak expression in *lasR* cells (Fig. 2). The second, seen most strongly for *phzA1* but also for *hcnA* (involved in phenazine and hydrogen cyanide synthesis, respectively), showed delayed but stronger expression by *lasR* mutant cells but weaker expression by the wild type (Fig. 2). These results revealed that wild-type cells were successfully performing quorum sensing, as they very strongly expressed *lasB* and also expressed *rhlA*. However, *phzA1* was notable for being largely turned off in the wild-type. The *lasR* mutant displayed the opposite phenotype, most strongly expressing genes that were weakly expressed by the wild type. Among the sampled quorum-regulated virulence genes, the wild-type and *lasR* strains thus showed distinct but complementary expression profiles, and the *lasR* profile was characterized by strong *phzA1* expression and pyocyanin production.

### LasR-independent expression requires the Rhl and PQS quorum-sensing systems

**Figure 3:**
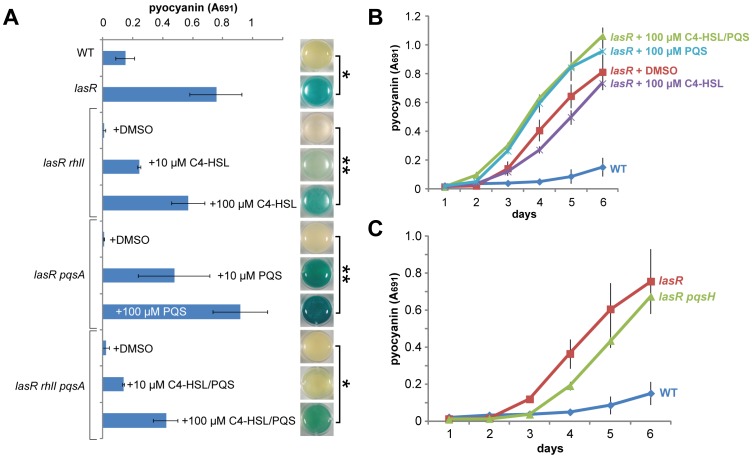
Pyocyanin production by stationary-phase *lasR* mutant cells requires Rhl and quinolone signaling. A. Pyocyanin quantification in the listed strains with or without the listed signaling molecules after 6 days of LB static culture at 25°C. The images to the right of the bar graph show the appearance of representative cultures. *, *p*<0.005; **, *p*<0.001. The differences between *lasR* and the 100 µM-complemented autoinducer-negative samples were not significant (0.04<*p*<0.33). B. Pyocyanin quantification in static LB cultures of the listed strains and conditions at 25°C. C. Pyocyanin quantification in static LB cultures of the listed strains at 25°C. All plots show the mean ± standard deviation of 3 biological replicates.

Previously reported LasR-independent quorum sensing in shaking culture required the Rhl quorum sensing system [15], in accord with its position in the quorum-sensing network (Fig. 1A). I thus tested whether the Rhl and PQS systems were also required for quorum expression in stationary-phase *lasR* cells. Indeed, additional deletion of *rhlR*, encoding the RhlR regulator, in a *lasR* background abolished the expression of all tested genes (Fig. 2). Similarly, pyocyanin production did not occur in *lasR rhlI* or *lasR pqsA* double mutants, which are unable to produce the Rhl autoinducer *N*-butyryl-l-homoserine lactone (C4-HSL) or 2-heptyl-4-quinolone (HHQ) and 2-heptyl-3-hydroxy-4-quinolone (PQS), respectively (Fig. 3A). Each of these double mutants could be complemented for pyocyanin production by exogenous addition of the appropriate autoinducer (C4-HSL or PQS), with stronger induction at 100 µM than at 10 µM (Fig. 3A). Consistent with these results, a triple *lasR rhlI pqsA* mutant required the addition of both autoinducers to restore pyocyanin production (Fig. 3A). Moreover, exogenous addition of PQS alone or in combination with C4-HSL to the *lasR* mutant accelerated pyocyanin production, while C4-HSL alone did not (Fig. 3B). This result is consistent with the idea that cellular RhlR levels are a limiting factor for LasR-independent pyocyanin production, as PQS signaling can stimulate *rhlR* transcription [27] and addition of constitutively expressed plasmid-borne *rhlR* greatly accelerated and increased pyocyanin production in a *lasR* mutant in shaking culture [15].

A *lasR pqsH* double mutant, which is unable to convert HHQ to PQS [28], [29], was able to produce pyocyanin (Fig. 3C), suggesting that HHQ is itself a signaling molecule that can functionally substitute for PQS to induce pyocyanin production under stationary-phase conditions. This result contrasts with a previous report [29], but the difference may be due to the different strain background, culture media and conditions used in this work.

It has been suggested that LasR-independent quorum sensing and pyocyanin production may occur through the PhoB-mediated phosphate starvation pathway [27] or use the newly discovered signaling molecule IQS, whose synthesis requires the AmbB protein [30]. To test whether pyocyanin production by stationary-phase *lasR* cells required either of these proteins in addition to Rhl and PQS quorum signaling, I constructed *lasR phoB* and *lasR ambB* double mutants and assayed them for pyocyanin production in static culture. Each of the double mutants produced pyocyanin indistinguishably from the *lasR* mutant (Fig. S3 in File S1), showing that neither of these pathways is required for LasR-independent overproduction of pyocyanin in stationary-phase culture.

### Repression by RsaL explains the different quorum profiles of wild-type and *lasR* cells

**Figure 4:**
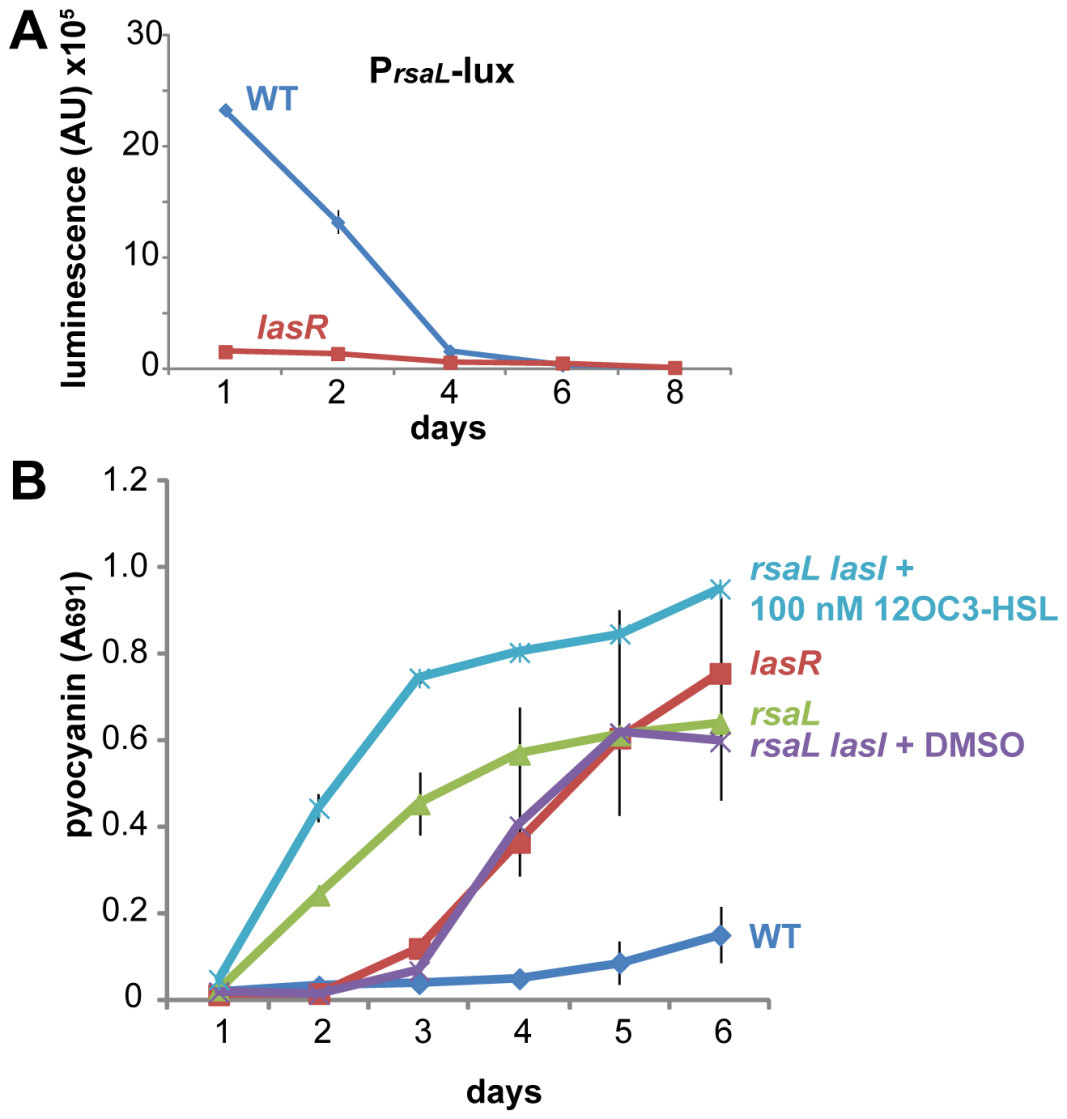
RsaL prevents pyocyanin production by stationary-phase *lasR* ^+^ cells. A. Transcription from the *rsaL* promoter in wild-type (PA14) and *lasR* mutant cells in static 25°C LB culture. B. Pyocyanin quantification in static LB cultures of the listed strains and conditions at 25°C. All plots show the mean ± standard deviation of 3 biological replicates.

The weak expression by wild-type cells of genes that were strongly expressed by the *lasR* mutant suggested that they might be under negative regulation. Notably, *phzA1* and *hcnA*, which displayed the strongest LasR-independent expression and the weakest expression by the wild type, are direct targets of negative regulation by RsaL, a repressor whose primary role is to provide negative homeostatic feedback to Las quorum sensing [31]. Meanwhile, *lasB* and *rhlA*, which are not under RsaL repression [31], were strongly expressed in the wild type. Because expression of *rsaL* is under LasR control [32], RsaL was an excellent candidate for a negative repressor that would be present in the wild type but absent in a *lasR* mutant. Indeed, stationary-phase *rsaL* expression in static culture was very strong in wild-type cells but quite weak in *lasR* cells (Fig. 4A). Presumably, the RsaL protein produced during the initial peak of expression in wild-type cells continues to stably bind its target DNA sequences [33], such as *phzA1*, in subsequent days, ensuring their continued repression.

If RsaL were responsible for repressing genes such as *phzA1* in otherwise quorum-active wild-type cells in stationary phase, inactivation of *rsaL* in a wild-type background would relieve this repression. Consistent with this hypothesis, an *rsaL* mutant in static culture displayed copious pyocyanin production that began significantly earlier than in a *lasR* mutant (Fig. 4B), suggesting that RsaL normally blocks pyocyanin production by the wild type. Deletion of *rsaL* also disrupts Las homeostasis, resulting in overabundance (25 µM, compared to 2 µM in the wild type) of the Las autoinducer *N*-3-oxo-dodecanoyl-l-homoserine lactone (3OC12-HSL) [31]. It was thus possible that high concentrations of 3OC12-HSL abetted the early production of pyocyanin. To correct for any such effect, I constructed an *rsaL lasI* double mutant unable to produce 3OC12-HSL and exogenously added a low (100 nM) concentration of 3OC12-HSL at the time of inoculation. The double mutant displayed 3OC12-HSL-dependent early pyocyanin production that was even stronger than that of the *rsaL* mutant (Fig. 4B), confirming that stationary-phase wild-type cells are capable of pyocyanin production but that it is repressed by the presence of the RsaL repressor. Therefore, expression of a specific set of quorum-regulated genes (including *phzA1* and *hcnA*) in *lasR* cells is caused by LasR-independent Rhl and PQS quorum-sensing activity in combination with deactivation of RsaL-mediated repression.

### *lasR* cells contribute pyocyanin in mixed culture even under conditions that permit cheating

**Figure 5:**
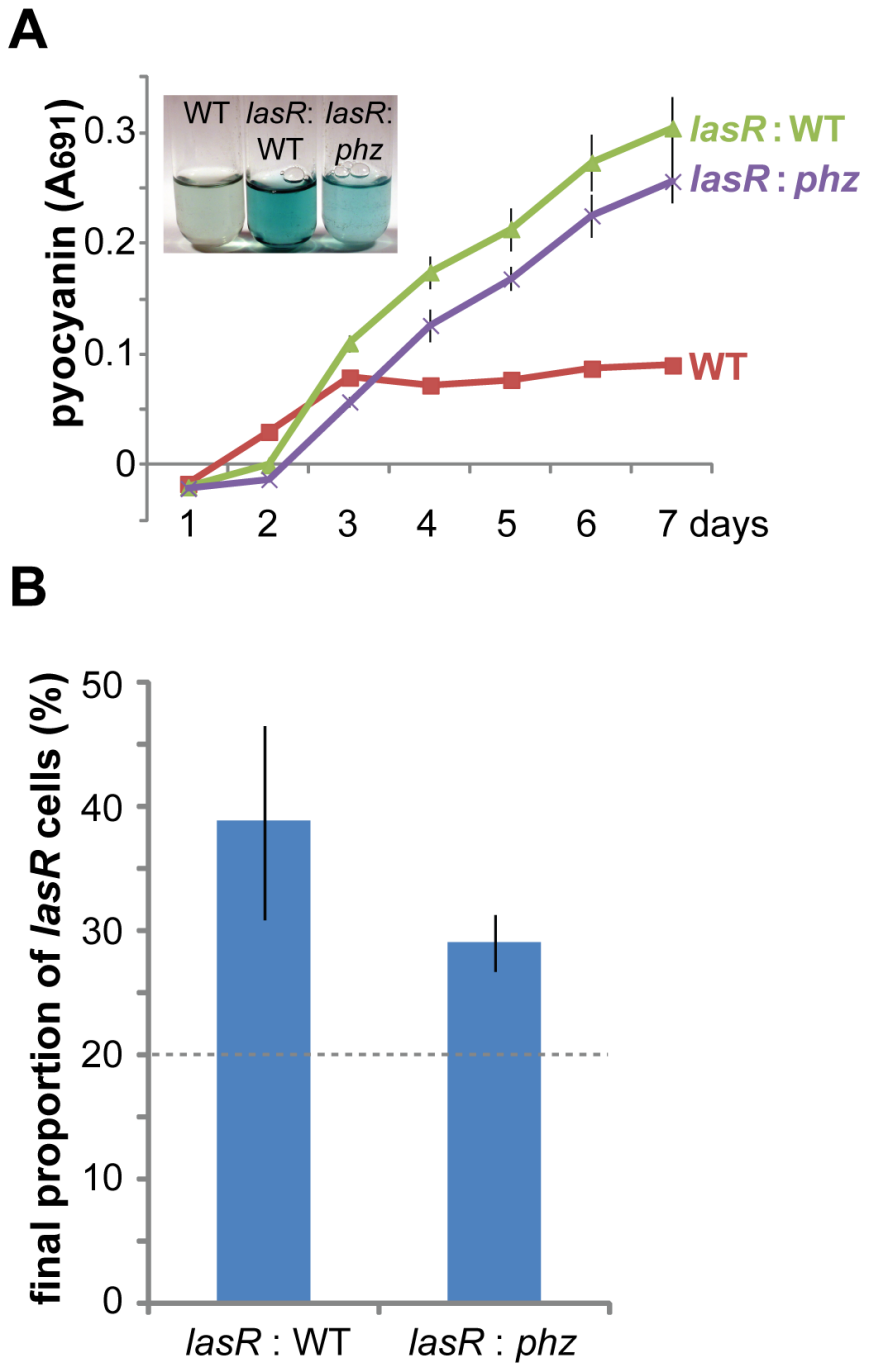
A *lasR* mutant contributes pyocyanin in a cheating mixture. Starting mixtures were 1:4 *lasR* mutant to PA14 or PA14 *phz*. All plots show the mean ± standard deviation of 3 biological replicates. A. Pyocyanin quantification in shaking M9-casein medium at 25°C. Inset shows appearance of cultures after 7 days of incubation. B. Relative proportions of *lasR* mutant cells in the indicated mixtures after 7 days of incubation. The difference between the two mixtures was not significant (*p* = 0.12).

A *lasR* mutant is a well-known example of a “cheater”. Typical cheating experiments use defined medium containing casein as the sole carbon source [12], [13], [34]. Because casein utilization requires quorum-regulated extracellular proteases such LasB, whose production in early phases of growth is induced by the Las system [35], a *lasR* mutant fails to grow on casein medium (Fig. S4 in File S1). When a wild-type strain is grown together with a *lasR* mutant, the *lasR* mutant benefits from the casein proteolysis performed by wild-type-derived LasB without the associated costs of producing quorum-regulated factors and thereby gains an advantage [12], [13]. In light of the distinct quorum-sensing profiles of stationary-phase wild-type and *lasR* cells, I hypothesized that *lasR* cells might be able to contribute quorum-regulated factors such as pyocyanin even while “cheating” with respect to nutrition. To test this hypothesis, I cultivated wild-type and *lasR* cells alone and in a 1∶4 mutant-to-wild-type mixture for several days in shaking liquid M9 medium with 1% casein, a typical cheating medium. As expected, the *lasR* mutant alone did not grow in this medium (Fig. S4 in File S1), while the wild-type grew (Fig. S4 in File S1) and produced some pyocyanin (Fig. 5A), indicating quorum sensing. The mixture of the two strains, however, produced much more pyocyanin than the wild-type alone (Fig. 5A), suggesting that the *lasR* mutant was contributing to pyocyanin production. To test this idea, I grew 1∶4 *lasR*-to-*phz* mixtures in which only the *lasR* mutant could contribute pyocyanin. Such mixtures produced only slightly less pyocyanin than mixtures with the wild-type and substantially more pyocyanin than the wild-type alone (Fig. 5A), confirming that the *lasR* mutant contributed the majority of pyocyanin in mixtures. In such mixtures, the relative *lasR* population increased from its initial 20% level to 30–40% after 7 days of culture (Fig. 5B), suggestive of a slight advantage gained by nutritional cheating. These results demonstrated that the *lasR* mutant cooperated with respect to pyocyanin production even under conditions (casein medium) that forced it to cheat with respect to LasB. Notably, pyocyanin production by the *lasR* cells was not detected until the third day of culture (Fig. 5A), explaining why this *lasR* phenotype is not seen in cheating assays when cultures are diluted every 1–2 days [12], [13].

### Clinical *lasR* isolates can overproduce pyocyanin

**Figure 6:**
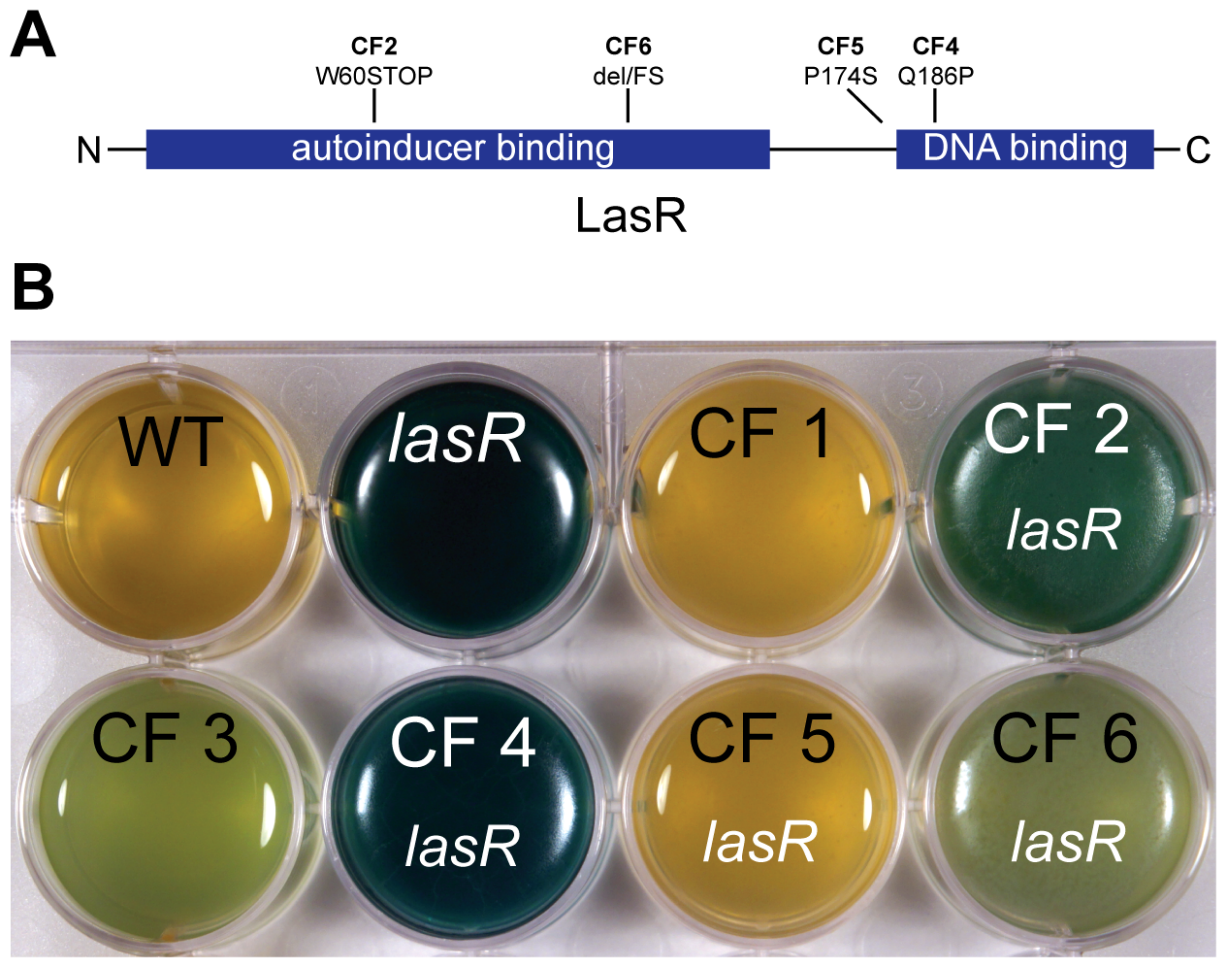
Clinical *lasR* isolates can overproduce pyocyanin. A. Locations of mutations in the LasR protein detected by sequencing. The mutations are in clinical isolates from Ref. 17. B. Appearance of static LB cultures of PA14 and *lasR* laboratory strains and of clinical isolates (CF1-6) [17] with and without *lasR* mutations. The cultures were grown for 12 days at 25°C to allow weaker phenotypes to develop.

The overproduction of pyocyanin by stationary-phase *lasR* cells in monoculture and in mixtures with wild-type cells raised the interesting possibility that *lasR* cells may overproduce pyocyanin in clinical infections, thereby increasing virulence. A relationship between the presence of *lasR* cells and high pyocyanin is at least suggested by separate studies associating high sputum pyocyanin [19] and *lasR* cell presence [17] with *P. aeruginosa* disease progression in cystic fibrosis patients. Assessing the relative contributions of *lasR* ^+^ and *lasR* cells to pyocyanin or virulence factor production in actual chronic human infections is very difficult, due to the spatial and genetic complexity of human lung infections. However, as a simple test of principle, I subjected a small set of clinical cystic fibrosis isolates that were wild type or mutant for *lasR* (Fig. 6A) to a static culture assay and looked for pyocyanin production. The *lasR* ^+^ strains exhibited minimal pyocyanin production, whereas *lasR* mutant strains showed widely varied production ranging from minimal to very strong (Fig. 6B). This variability may reflect differences in the severity of the different *lasR* mutant alleles (Fig. 6A) and other mutations in the genetic backgrounds of these strains that modulate pyocyanin production. Nonetheless, these results demonstrate that some clinical *lasR* mutant strains have the ability to overproduce pyocyanin under stationary-phase conditions where *lasR* ^+^ isolates cannot.

## Discussion

This work shows that the quorum response by *lasR* mutants in slow-growth or stationary-phase conditions is distinct from the wild-type response and is characterized by strong expression of virulence factor genes that are repressed in wild-type cells by RsaL. For example, the pattern of low pyocyanin production by wild-type and high production by *lasR* cells in static stationary-phase culture is a reversal of the pattern seen for cells growing exponentially in shaking culture, showing that “typical” lab conditions uncover only part of the full range of cell behaviors. Experiments conducted in shaking culture for 24 hours showed that *lasR* cells could manifest a quorum response [15], but did not reveal the distinctions between the wild-type and *lasR* stationary-phase phenotypes that develop after longer-term culture under slow-growth conditions.

Stationary-phase phenotypes are highly relevant for bacterial physiology in natural settings, including within infective biofilms [20], [21], [22]. The relative metabolic inactivity of some biofilm-embedded cells is one mechanism of resistance against killing by host defenses or by antibiotics [24]. Moreover, host environments like the cystic fibrosis lung contain hypoxic niches that slow bacterial growth [36], [37]. It is therefore likely that many *P. aeruginosa* cells in long-term infections are enacting stationary-phase behaviors. In such conditions, the presence of *lasR* mutants within the *P. aeruginosa* population may permit the expression of important virulence factors such as pyocyanin that are repressed by RsaL in *lasR* ^+^ cells, thus expanding the range of phenotypes available to the total population. In this way, niches containing *lasR* cells could make a key contribution to virulence.

If repression by RsaL prevents *lasR* ^+^ cells from producing important virulence factors, why are mutations in *rsaL* not commonly isolated in clinical samples from chronic infections? One likely reason is because of the homeostatic function of RsaL in the normal quorum response. Cells lacking RsaL function display constitutive overproduction of quorum-regulated factors [38], perhaps making an *rsaL* cell population less competitive than wild-type cells under faster-growth conditions in the same way that wild-type cells can be cheated on by *lasR* cells. In contrast, a *lasR* mutant can be competitive under fast-growth conditions (as illustrated by its well-known cheating behavior) before overproducing a more narrowly defined set of quorum-regulated factors specifically during stationary phase. This fine tuning is made possible by a combination of three features of the quorum-sensing regulatory circuit: first, RsaL is under LasR control and thus is not produced in a *lasR* mutant [32]; second, RsaL has many other targets in addition to its homeostatic regulation of *lasI* [31]; and third, the Rhl and PQS systems, which are normally activated by LasR, can also self-activate in a *lasR* mutant [15], [16].

The distinct contributions of *lasR* ^+^ and *lasR* cells in a mixture allows them to collaborate to produce otherwise inaccessible phenotypes. This is seen most clearly in casein medium, where the *lasR* ^+^ cells secrete LasB to break down casein and feed the *lasR* cells, and the *lasR* cells in turn produce high levels of pyocyanin. It is conceivable that such a division of labor, where *lasR* cells overproduce pyocyanin and other virulence factors, may have a role in host infection. In this scenario, slow-growing or stationary-phase *lasR* cells within an infecting population might continually produce pyocyanin under conditions where *lasR* ^+^ cells do not. Overproduction of pyocyanin by some clinical *lasR* isolates under stationary-phase laboratory conditions suggests that they may do likewise in an infection setting, in accord with the findings that *lasR* strains [17] and high sputum pyocyanin [19] are both correlated with disease progression in cystic fibrosis patients. One corollary of this idea is that treatment strategies based on strong pharmacological inhibition of LasR (thus making cells functionally null for LasR) may in fact increase pyocyanin production by *lasR* ^+^ cells in stationary phase.

## Supporting Information

File S1 **This file contains Tables S1–S3 and Figure S1–S4.** Table S1, *Escherichia coli* strains used in this study. Table S2, Plasmids used in this study. Table S3, Primer sequences used in this study. Figure S1, Pyocyanin production in static culture. Figure S2, Pyocyanin production by *lasR* cells after overnight regrowth. Figure S3, LasR-independent pyocyanin production does not depend on AmbB or PhoB. Figure S4, Growth of wild-type PA14 or lasR cells in M9 medium with added casamino acids or casein.
(PDF)

## References

[1] Driscoll JA, Brody SL, Kollef MH (2007) The epidemiology, pathogenesis and treatment of *Pseudomonas aeruginosa* infections. Drugs 67: 351–368. 17335295 10.2165/00003495-200767030-00003

[2] Lyczak JB, Cannon CL, Pier GB (2002) Lung infections associated with cystic fibrosis. Clin Microbiol Rev 15: 194–222. 11932230 10.1128/CMR.15.2.194-222.2002 PMC118069

[3] Rutherford ST, Bassler BL (2012) Bacterial quorum sensing: its role in virulence and possibilities for its control. Cold Spring Harb Perspect Med 2.. 10.1101/cshperspect.a012427 PMC3543102 23125205

[4] Dietrich LE, Price-Whelan A, Petersen A, Whiteley M, Newman DK (2006) The phenazine pyocyanin is a terminal signalling factor in the quorum sensing network of *Pseudomonas aeruginosa* . Mol Microbiol 61: 1308–1321. 16879411 10.1111/j.1365-2958.2006.05306.x

[5] D'Argenio DA, Wu M, Hoffman LR, Kulasekara HD, Deziel E, et al (2007) Growth phenotypes of *Pseudomonas aeruginosa* lasR mutants adapted to the airways of cystic fibrosis patients. Mol Microbiol 64: 512–533. 17493132 10.1111/j.1365-2958.2007.05678.x PMC2742308

[6] Hentzer M, Wu H, Andersen JB, Riedel K, Rasmussen TB, et al (2003) Attenuation of *Pseudomonas aeruginosa* virulence by quorum sensing inhibitors. EMBO J 22: 3803–3815. 12881415 10.1093/emboj/cdg366 PMC169039

[7] Pesci EC, Pearson JP, Seed PC, Iglewski BH (1997) Regulation of *las* and *rhl* quorum sensing in *Pseudomonas aeruginosa* . J Bacteriol 179: 3127–3132. 9150205 10.1128/jb.179.10.3127-3132.1997 PMC179088

[8] Schuster M, Lostroh CP, Ogi T, Greenberg EP (2003) Identification, timing, and signal specificity of *Pseudomonas aeruginosa* quorum-controlled genes: a transcriptome analysis. J Bacteriol 185: 2066–2079. 12644476 10.1128/JB.185.7.2066-2079.2003 PMC151497

[9] Smith RS, Iglewski BH (2003) *P. aeruginosa* quorum-sensing systems and virulence. Curr Opin Microbiol 6: 56–60. 12615220 10.1016/s1369-5274(03)00008-0

[10] Wagner VE, Bushnell D, Passador L, Brooks AI, Iglewski BH (2003) Microarray analysis of *Pseudomonas aeruginosa* quorum-sensing regulons: effects of growth phase and environment. J Bacteriol 185: 2080–2095. 12644477 10.1128/JB.185.7.2080-2095.2003 PMC151498

[11] Rumbaugh KP, Diggle SP, Watters CM, Ross-Gillespie A, Griffin AS, et al (2009) Quorum sensing and the social evolution of bacterial virulence. Curr Biol 19: 341–345. 19230668 10.1016/j.cub.2009.01.050

[12] Diggle SP, Griffin AS, Campbell GS, West SA (2007) Cooperation and conflict in quorum-sensing bacterial populations. Nature 450: 411–414. 18004383 10.1038/nature06279

[13] Sandoz KM, Mitzimberg SM, Schuster M (2007) Social cheating in *Pseudomonas aeruginosa* quorum sensing. Proc Natl Acad Sci U S A 104: 15876–15881. 17898171 10.1073/pnas.0705653104 PMC2000394

[14] Smith EE, Buckley DG, Wu Z, Saenphimmachak C, Hoffman LR, et al (2006) Genetic adaptation by *Pseudomonas aeruginosa* to the airways of cystic fibrosis patients. Proc Natl Acad Sci U S A 103: 8487–8492. 16687478 10.1073/pnas.0602138103 PMC1482519

[15] Dekimpe V, Deziel E (2009) Revisiting the quorum-sensing hierarchy in *Pseudomonas aeruginosa*: the transcriptional regulator RhlR regulates LasR-specific factors. Microbiology 155: 712–723. 19246742 10.1099/mic.0.022764-0

[16] Diggle SP, Winzer K, Chhabra SR, Worrall KE, Camara M, et al (2003) The *Pseudomonas aeruginosa* quinolone signal molecule overcomes the cell density-dependency of the quorum sensing hierarchy, regulates rhl-dependent genes at the onset of stationary phase and can be produced in the absence of LasR. Mol Microbiol 50: 29–43. 14507361 10.1046/j.1365-2958.2003.03672.x

[17] Hoffman LR, Kulasekara HD, Emerson J, Houston LS, Burns JL, et al (2009) *Pseudomonas aeruginosa lasR* mutants are associated with cystic fibrosis lung disease progression. J Cyst Fibros 8: 66–70. 18974024 10.1016/j.jcf.2008.09.006 PMC2631641

[18] Rada B, Leto TL (2013) Pyocyanin effects on respiratory epithelium: relevance in *Pseudomonas aeruginosa* airway infections. Trends Microbiol 21: 73–81. 23140890 10.1016/j.tim.2012.10.004 PMC3565070

[19] Hunter RC, Klepac-Ceraj V, Lorenzi MM, Grotzinger H, Martin TR, et al (2012) Phenazine content in the cystic fibrosis respiratory tract negatively correlates with lung function and microbial complexity. Am J Respir Cell Mol Biol 47: 738–745. 22865623 10.1165/rcmb.2012-0088OC

[20] Bjarnsholt T, Alhede M, Alhede M, Eickhardt-Sørensen SR, Moser C, et al (2013) The in vivo biofilm. Trends in microbiology 21: 466–474. 23827084 10.1016/j.tim.2013.06.002

[21] Fux CA, Costerton JW, Stewart PS, Stoodley P (2005) Survival strategies of infectious biofilms. Trends Microbiol 13: 34–40. 15639630 10.1016/j.tim.2004.11.010

[22] Foley I, Marsh P, Wellington EM, Smith AW, Brown MR (1999) General stress response master regulator *rpoS* is expressed in human infection: a possible role in chronicity. J Antimicrob Chemother 43: 164–165. 10381122 10.1093/jac/43.1.164

[23] Xu KD, Franklin MJ, Park CH, McFeters GA, Stewart PS (2001) Gene expression and protein levels of the stationary phase sigma factor, RpoS, in continuously-fed *Pseudomonas aeruginosa* biofilms. FEMS Microbiol Lett 199: 67–71. 11356569 10.1111/j.1574-6968.2001.tb10652.x

[24] Mah TF, O'Toole GA (2001) Mechanisms of biofilm resistance to antimicrobial agents. Trends Microbiol 9: 34–39. 11166241 10.1016/s0966-842x(00)01913-2

[25] Palmer KL, Aye LM, Whiteley M (2007) Nutritional cues control *Pseudomonas aeruginosa* multicellular behavior in cystic fibrosis sputum. J Bacteriol 189: 8079–8087. 17873029 10.1128/JB.01138-07 PMC2168676

[26] Navarro Llorens JM, Tormo A, Martinez-Garcia E (2010) Stationary phase in gram-negative bacteria. FEMS Microbiol Rev 34: 476–495. 20236330 10.1111/j.1574-6976.2010.00213.x

[27] Jensen V, Lons D, Zaoui C, Bredenbruch F, Meissner A, et al (2006) RhlR expression in *Pseudomonas aeruginosa* is modulated by the *Pseudomonas* quinolone signal via PhoB-dependent and -independent pathways. J Bacteriol 188: 8601–8606. 17028277 10.1128/JB.01378-06 PMC1698233

[28] Deziel E, Lepine F, Milot S, He J, Mindrinos MN, et al (2004) Analysis of *Pseudomonas aeruginosa* 4-hydroxy-2-alkylquinolines (HAQs) reveals a role for 4-hydroxy-2-heptylquinoline in cell-to-cell communication. Proc Natl Acad Sci U S A 101: 1339–1344. 14739337 10.1073/pnas.0307694100 PMC337054

[29] Gallagher LA, McKnight SL, Kuznetsova MS, Pesci EC, Manoil C (2002) Functions required for extracellular quinolone signaling by *Pseudomonas aeruginosa* . J Bacteriol 184: 6472–6480. 12426334 10.1128/JB.184.23.6472-6480.2002 PMC135424

[30] Lee J, Wu J, Deng Y, Wang J, Wang C, et al (2013) A cell-cell communication signal integrates quorum sensing and stress response. Nat Chem Biol. 10.1038/nchembio.1225 23542643

[31] Rampioni G, Schuster M, Greenberg EP, Bertani I, Grasso M, et al (2007) RsaL provides quorum sensing homeostasis and functions as a global regulator of gene expression in *Pseudomonas aeruginosa* . Mol Microbiol 66: 1557–1565. 18045385 10.1111/j.1365-2958.2007.06029.x

[32] de Kievit T, Seed PC, Nezezon J, Passador L, Iglewski BH (1999) RsaL, a novel repressor of virulence gene expression in *Pseudomonas aeruginosa* . J Bacteriol 181: 2175–2184. 10094696 10.1128/jb.181.7.2175-2184.1999 PMC93631

[33] Rampioni G, Polticelli F, Bertani I, Righetti K, Venturi V, et al (2007) The *Pseudomonas* quorum-sensing regulator RsaL belongs to the tetrahelical superclass of H-T-H proteins. J Bacteriol 189: 1922–1930. 17172347 10.1128/JB.01552-06 PMC1855778

[34] Dandekar AA, Chugani S, Greenberg EP (2012) Bacterial quorum sensing and metabolic incentives to cooperate. Science 338: 264–266. 23066081 10.1126/science.1227289 PMC3587168

[35] Van Delden C, Pesci EC, Pearson JP, Iglewski BH (1998) Starvation selection restores elastase and rhamnolipid production in a *Pseudomonas aeruginosa* quorum-sensing mutant. Infect Immun 66: 4499–4502. 9712807 10.1128/iai.66.9.4499-4502.1998 PMC108545

[36] Alvarez-Ortega C, Harwood CS (2007) Responses of *Pseudomonas aeruginosa* to low oxygen indicate that growth in the cystic fibrosis lung is by aerobic respiration. Mol Microbiol 65: 153–165. 17581126 10.1111/j.1365-2958.2007.05772.x PMC4157922

[37] Worlitzsch D, Tarran R, Ulrich M, Schwab U, Cekici A, et al (2002) Effects of reduced mucus oxygen concentration in airway *Pseudomonas* infections of cystic fibrosis patients. J Clin Invest 109: 317–325. 11827991 10.1172/JCI13870 PMC150856

[38] Rampioni G, Schuster M, Greenberg EP, Zennaro E, Leoni L (2009) Contribution of the RsaL global regulator to *Pseudomonas aeruginosa* virulence and biofilm formation. FEMS Microbiol Lett 301: 210–217. 19878323 10.1111/j.1574-6968.2009.01817.x

