# Supplementary material for: Stationary Phase-Specific Virulence Factor Overproduction by a lasR Mutant of Pseudomonas aeruginosa
Source: PLoS One. 2014 Feb 12;9(2):e88743. doi: 10.1371/journal.pone.0088743 (PMC3923063; doi:10.1371/journal.pone.0088743)
Supplement: File S1 — This file contains Tables S1–S3 and Figure S1–S4. Table S1, Escherichia coli strains used in this study. Table S2, Plasmids used in this study. Table S3, Primer sequences used in this study. Figure S1, Pyocyanin production in static culture. Figure S2, Pyocyanin production by lasR cells after overnight regrowth. Figure S3, LasR-independent pyocyanin production does not depend on AmbB or PhoB. Figure S4, Growth of wild-type PA14 or lasR cells in M9 medium with added casamino acids or casein. (PDF) [file pone.0088743.s001.pdf]

Supporting Information

Cabeen | Stationary phase-specific virulence factor overproduction by a *lasR* mutant of *Pseudomonas aeruginosa*

Table S1. ***Escherichia coli*** **strains used in this study**

Strain		Relevant genotype or description	Source or reference
MTC27		SM10 (F- *endA1 hsdR17 supE44 thi-1* λ-*recA1 gyrA96 relA1*)	[1]
MTC257		SM10 / pCTX-1-aac	This study
MTC296		SM10 / pEXG2-ΔlasR	This study
MTC410		SM10 / pEXG2-ΔpqsA	This study
MTC413		SM10 / pEXG2-ΔrhlI	This study
MTC417		SM10 / pEXG2-ΔrhlR	This study
MTC467		SM10 / pEXG2-ΔrsaL	This study
MTC488		SM10 / pEXG2-ΔrsaLΔlasI	This study
MTC708		SM10 / pCTX-1-P_{lasB}-lux	This study
MTC709		SM10 / pCTX-1-P_{phzA1}-lux	This study
MTC716		SM10 / pCTX-1-P_{hcnA}-lux	This study
MTC718		SM10 / pCTX-1-P_{rhlA}-lux	This study
MTC721		SM10 / pCTX-1-P_{rsaL}-lux	This study
MTC764		SM10 / pCTX-1-lux	This study
MTC808		SM10 / pEXG2-ΔambB	This study
MTC809		SM10 / pEXG2-ΔphoB	This study

Table S2. Plasmids used in this study.

Plasmid	Description	Source or reference
pCTX-1	mini-CTX-1, integrative tet^R plasmid for *P. aeruginosa*	[2]
pCTX-1-aacC1	CTX-1 containing the *aacC1* gene, conferring $gent^R$	This study
pCTX-1-lux	CTX-1 reporter construct containing *luxA-E*	[3]
pCTX-1-P_{hcnA}-lux	CTX-1 lux reporter driven from *hcnA* promoter	This study
pCTX-1-P_{lasB}-lux	CTX-1 lux reporter driven from *lasB* promoter	This study
pCTX-1-P_{phzA1}-lux	CTX-1 lux reporter driven from *phzA1* promoter	This study
pCTX-1-P_{rhlA}-lux	CTX-1 lux reporter driven from *phzA2* promoter	This study
pCTX-1-P_{rsaL}-lux	CTX-1 lux reporter driven from *rsaL* promoter	This study
pEXG2	Integrating suicide plasmid for *P. aeruginosa*, $gent^R$, with sucrose counterselection	[4]
pEXG2-ΔambB	EXG2 containing flanking sequences of *ambB*	This study
pEXG2-ΔlasR	EXG2 containing flanking sequences of *lasR*	This study

pEXG2-ΔrhlI	EXG2 containing flanking sequences of *rhlI*	This study
pEXG2-ΔrhlR	EXG2 containing flanking sequences of *rhlR*	This study
pEXG2-ΔrsaL	EXG2 containing flanking sequences of *rsaL*	This study
pEXG2-ΔrsaLΔlasI	EXG2 containing flanking sequences of the *rsaL-lasI* region	This study
pEXG2-ΔphoB	EXG2 containing flanking sequences of *phoB*	This study
pEXG2-ΔpqsA	EXG2 containing flanking sequences of *pqsA*	This study

Table S3. Primer sequences used in this study.

**Primer No.**	**Sequence (5'-3')**
82	GACGGTATCGATAAGCTTGATATCGAATTCGATGATCGTCCACATGGCCC
86	GACGGTATCGATAAGCTTGATATCGAATTCCGTTACACATTTCCGTAACCCG
94	GACGGTATCGATAAGCTTGATATCGAATTCCGTTTCGACACCGGAAACC
120	TCTAGAACTAGTGGATCCCCCGGGCTGCAGGAATTCCGAATTGACATAAGCCTGTTCGG
121	CCCCCTCGAGGTCGACGGTATCGATAAGCTTCTCGGCTTGAACGAATTGTTAGG
128	CAGAGAGTAAAGCGCTACGTTCTTCTTAAAC
129	TGCGCACCCGTGGAAATTAATTAAGGTACCGAATTCAACTGGAAAAGTGGCTATGTC
131	ACGTAGCGCTTTACTCTCTGATCTTGCCTCTC
132	TTATACGAGCCGGAAGCATAAATGTAAAGCAAGCTTGGACTGAGTGCGTCATAAC
134	TTATACGAGCCGGAAGCATAAATGTAAAGCAAGCTTCTACGAGAACGCCTTCATC
136	TGCGCACCCGTGGAAATTAATTAAGGTACCGAATTCTTGGCATTGAGTTCGATGC
173	TTATACGAGCCGGAAGCATAAATGTAAAGCAAGCTTGCGTCTCGACCTGGCGATGC
178	CGAACTTCTTTGCAGTAAGCCCTGATCG
179	TGCGCACCCGTGGAAATTAATTAAGGTACCGAATTCCGAACTGCAACGCTTTCTC
180	GCTTACTGCAAAGAAGTTCGACGCGCCG
181	TTATACGAGCCGGAAGCATAAATGTAAAGCAAGCTTGTTTCGCTGCACAGGTAGG
182	CTGACGACCTGACCAAGTCCCCGTGTCG
183	TGCGCACCCGTGGAAATTAATTAAGGTACCGAATTCGAACGAGGCTCGCGATTG
184	GGACTTGGTCAGGTCGTCAGCCGTTTCG
185	TTATACGAGCCGGAAGCATAAATGTAAAGCAAGCTTGCTACAGAGGGTGATCGGC
186	TCAACATGCCGACAGAACGTTCCCTCTTCAGCG

187	TGCGCACCCGTGGAAATTAATTAAGGTACCGAATTCTCGGCAGGATCGC CCAGTG
188	ACGTTCTGTCGGCATGTTGATTCAGGCTGTGG
189	TTATACGAGCCGGAAGCATAAATGTAAAGCAAGCTTCCTTGACGTCGCC TACCTGG
214	TGCGCACCCGTGGAAATTAATTAAGGTACCGAATTCCTACGAGAACGCC TTCATC
225	GATCAGAGCACCGACCTGAGAGGCAAGA
226	CTCAGGTCGGTGCTCTGATCTTTTCGGAC
227	CAGGTCCCCGCCGACCTGAGAGGCAAGA
232	CTCAGGTCGGCGGGGACCTGTCGGCTCG
353	TTTACCGGCAGATTTCTAAAGAAGAATTGGGGATTCTCCGAGAGGGCTC TCCAG
356	TTTACCGGCAGATTTCTAAAGAAGAATTGGGGATTCAACAGGCAAACAG CTATCGC
357	TTTACCGGCAGATTTCTAAAGAAGAATTGGGGATCCCAGTTCTCCTGGTT TTTTCAGGCC
358	GACGGTATCGATAAGCTTGATATCGAATTC GGTTCGTTTTCCCGCATATGCC
359	TTTACCGGCAGATTTCTAAAGAAGAATTGGGGATTCTGCCCTTTCATCCG TGAGAGAG
360	GACGGTATCGATAAGCTTGATATCGAATTCCGCGCCGACCAATTTGTACG
361	TTTACCGGCAGATTTCTAAAGAAGAATTGGGGATTCTGCTCTGATCTTTT CGGACGTTTC
370	GCCACGCGCCCGCTCTCCCCCTTCCGAG
371	TGCGCACCCGTGGAAATTAATTAAGGTACCGAATTCCCGCACATCCTCG GGATC
372	GGGGAGAGCGGGCGCGTGGCTATTTCGG
373	TTATACGAGCCGGAAGCATAAATGTAAAGCAAGCTTTAGAAACCCTGCC CGGCG
376	CTCTTGGTGGGGTCTTGCCTCGGGTCGATC
377	TGCGCACCCGTGGAAATTAATTAAGGTACCGAATTCGGCCTGGCGATCTT CCTCG
378	AGGCAAGACCCCACCAAGAGCTGACCCCG
379	TTATACGAGCCGGAAGCATAAATGTAAAGCAAGCTTCCTTGAAGCGCGG ATGGC

NB: The listed primer sequences include 5' overlaps for isothermal assembly and/or stitch PCR. The 3' end is complementary to the target genomic sequence.

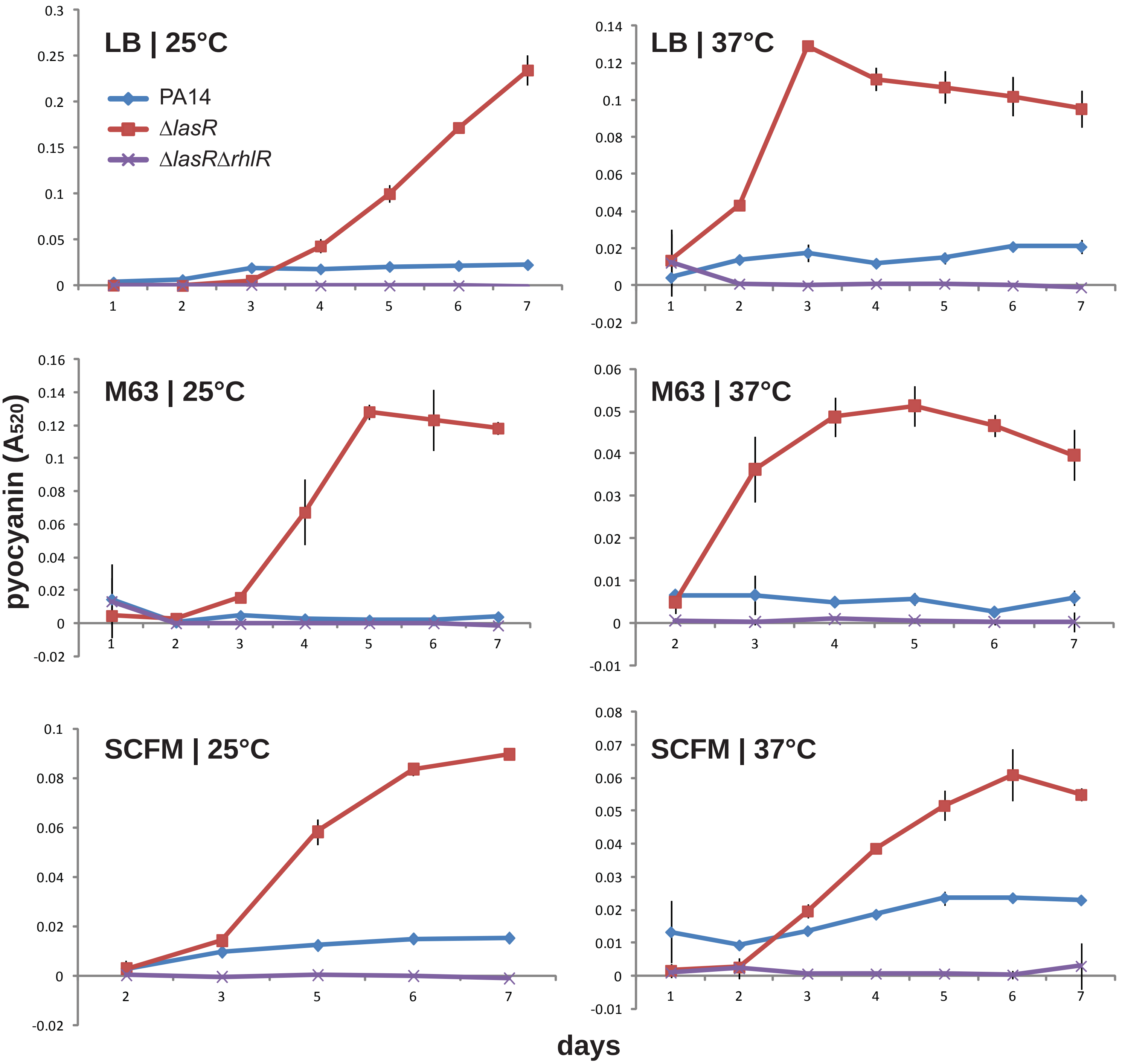

Figure S1

Figure S1. Pyocyanin production in static culture. Pyocyanin production by wild-type PA14 (MTC1), *lasR* (MTC390), or *lasR rhlR* (MTC626) strains was monitored over several days of growth in static liquid cultures in the indicated media at 25°C or 37°C as indicated. LB, Luria-Bertani medium. M63, M63 minimal medium. SCFM, synthetic cystic fibrosis medium (Palmer et al., 2007). Mean values (n = 3) are plotted, and error bars indicate standard deviation.

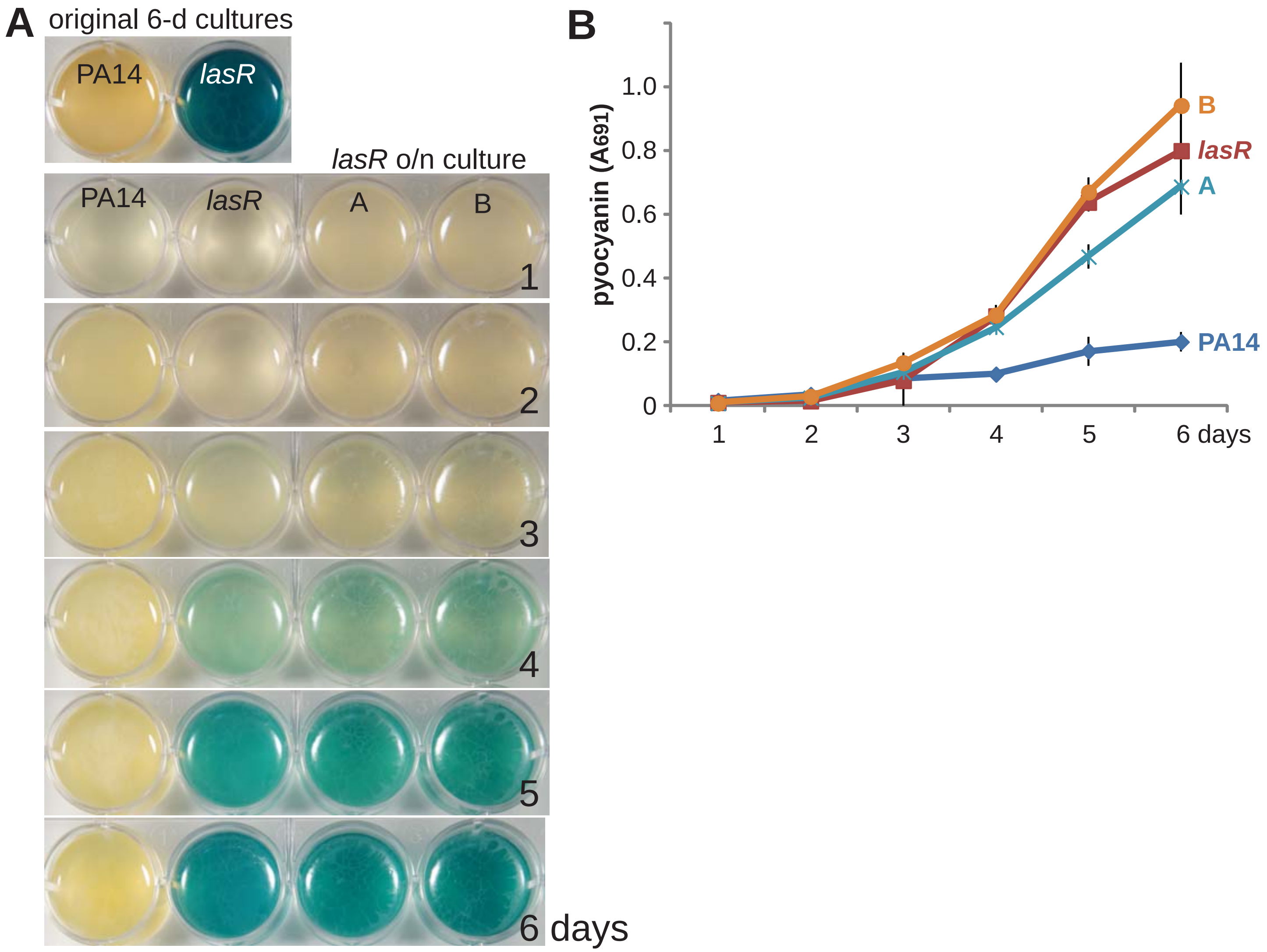

Figure S2

Figure S2. Pyocyanin production by *lasR* cells after overnight regrowth. A. Representative images of 6-day-old *lasR* (MTC390) cultures (top) used for the experiment. The blue 6-day-old cultures (*n* = 2) were used to inoculate fresh LB, and the cultures were grown overnight at 37°C in a tube roller. The regrown *lasR* cultures were then diluted with LB to an OD_{600} of 1.0 and inoculated into fresh 4-ml static LB culture (3 replicates) at an initial OD_{600} of 0.01 simultaneously with PA14 and *lasR* cultures grown from a freshly streaked plate taken from the freezer stock. The static cultures were grown at 25°C and photographed daily. **B.** Cultures were also sampled daily to quantify pyocyanin. A and B refer to the regrown cultures shown in (A). Data represent the mean of three replicate cultures, and error bars show standard deviation.

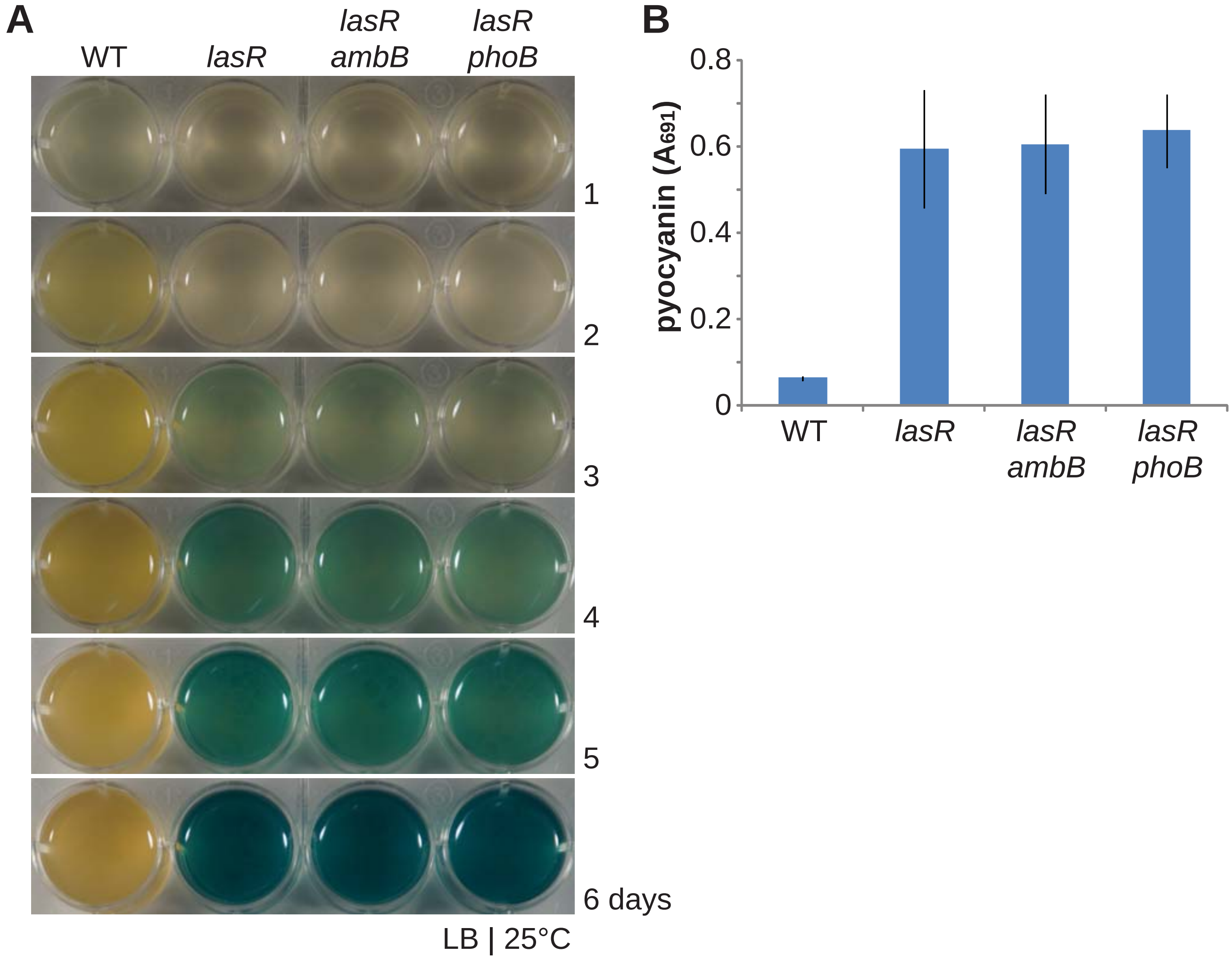

Figure S3

Figure S3. LasR-independent pyocyanin production does not depend on AmbB or PhoB. A. Wild-type PA14 (WT; MTC1), *lasR* (MTC390), *lasR ambB* (MTC838), and *lasR phoB* (MTC842) were grown in 4-ml static LB cultures for 6 days and photographed daily. The presence of *ambB* or *phoB* deletions in addition to the *lasR* deletion did not visibly change the onset of pyocyanin production. **B.** Quantification of pyocyanin by the cultures in (A) after 6 days. Data represent the mean values from 3 independent cultures, and error bars show standard deviation.

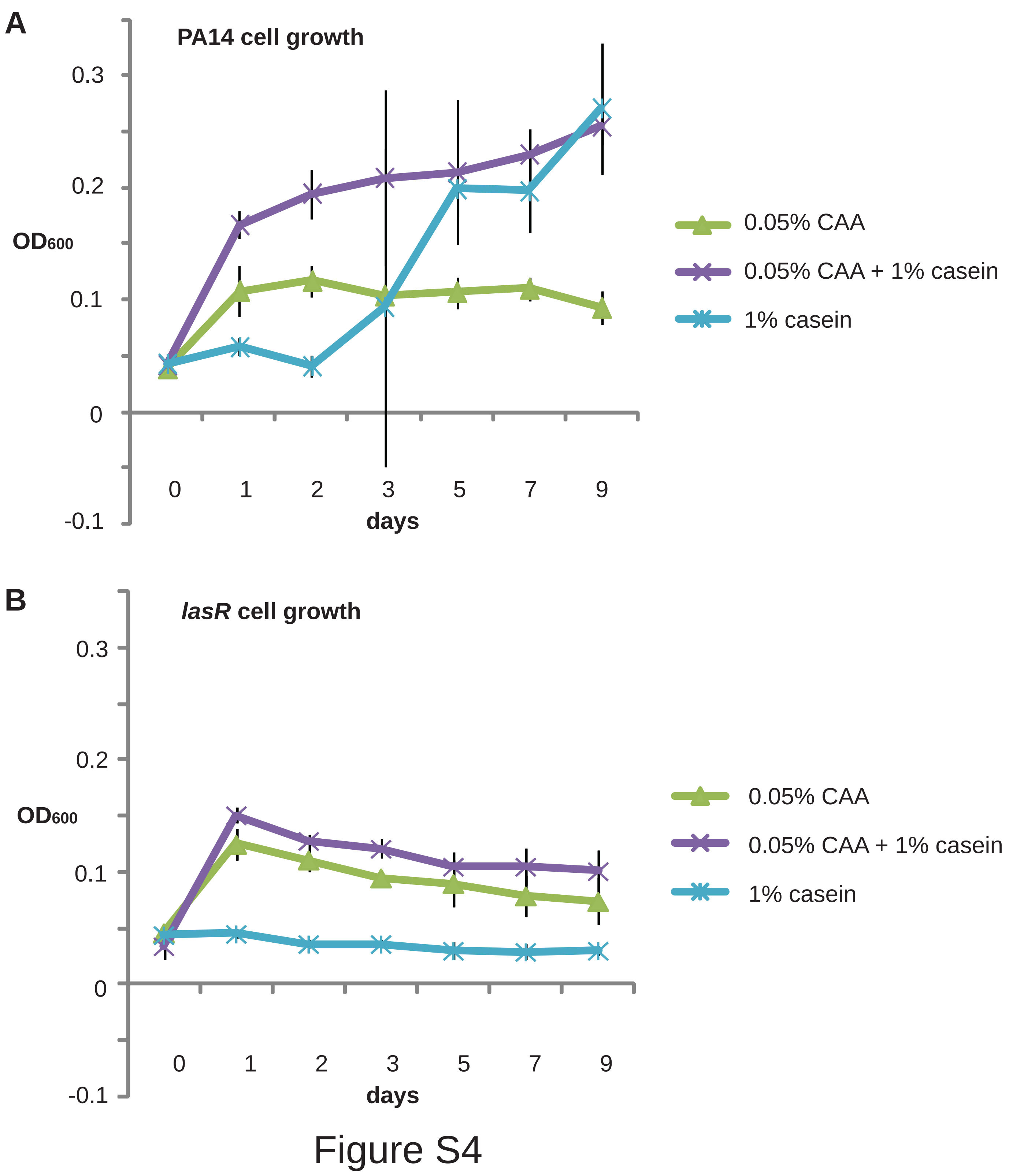

Figure S4. Growth of wild-type PA14 or lasR cells in M9 medium with added casamino acids or casein. A. Cell growth (OD_{600}) of wild-type PA14 (MTC1) cells in M9 minimal medium containing casamino acids (CAA) and/or casein at the indicated concentrations as the sole carbon source(s). Inocula from LB starter cultures were washed with M9 salts before inoculation, and the cells were grown in shaking culture at 25°C. The large standard deviation at day 3 when growing on casein is likely caused by inter-culture variation in the onset time of casein utilization. The wild-type cells were able to use casein alone, and the additional presence of casamino acids appeared to abet the transition to casein utilization. **B.** Cell growth of lasR cultures (MTC390) as in (A). Growth did not occur in the presence of casein alone, and the presence of both casein and casamino acids did not support growth beyond the level permitted by casamino acids alone.

Modes of strain construction

P. aeruginosa strains

MTC390

MTC1 was mated with MTC296: 50 µL of an overnight LB culture of MTC296 was spot-dried on an LB plate, and 100 µL of an overnight LB culture of MTC1 was subsequently dried on top of the first spot. The plate was incubated at 37°C overnight, and the resulting colony was scraped up with a sterile loop and resuspended in 500 µL sterile phosphate-buffered saline (PBS). An aliquot (typically 100 µL) of the suspension was spread on LB agar containing 75 µg/ml gentamycin and 25 µg/ml irgasan to select for *P. aeruginosa* transformants with integrated pEXG2-ΔlasR plasmid. Several of the resulting colonies were then inoculated into plain LB and grown at 37°C for 4-6 hours, to accumulate second crossovers. Aliquots (typically 25 µL and 100 µL) of the LB culture were then spread on LB plates containing 6% sucrose, to select against the plasmid. A number of the sucrose-resistant colonies arising were then patched on LB and LB with 20 µg/mL gentamycin. At least 2 sucrose-resustant, gent-sensitive clones were then streaked for single colonies, checked by PCR for presence of the desired deletion, and frozen at -80°C in 25% glycerol.

MTC498

Constructed like MTC390, but MTC1 was mated with MTC467.

MTC500

Constructed like MTC390, but MTC1 was mated with MTC488.

MTC537

Constructed like MTC390, but MTC1 was mated with MTC410.

MTC556

Constructed like MTC390, but MTC537 was mated with MTC413.

MTC625

Constructed like MTC390, but MTC390 was mated with MTC413.

MTC626

Constructed like MTC390, but MTC390 was mated with MTC417.

MTC628

Constructed like MTC390, but MTC390 was mated with MTC410.

MTC637

MTC390 was mated with MTC257 as described above, and a 10-µL aliquot of the PBS-resuspended mating mix was spread on LB plates with 75 µg/mL tetracycline and 25 µg/mL irgasan to select for *P. aeruginosa* transformants. At least 2 colonies were then re-streaked for single colonies on LB-gent (20 µg/mL), confirming that the resulting strain was gentamycin-resistant. Gentamycin-resistant colonies were grown overnight in LB at 37°C and stored in 25% glycerol at -80°C.

MTC723

MTC1 was mated with MTC708 as described above, and a 10-µL aliquot of the PBS-resuspended mating mix was spread on LB plates with 75 µg/mL tetracycline and 25 µg/mL irgasan to select for *P. aeruginosa* transformants. At least 2 colonies were then re-streaked for single colonies on LB-tet (25 µg/mL), grown in LB overnight at 37°C, and stored in 25% glycerol at -80°C.

MTC 725

Constructed like MTC723, but MTC1 was mated with MTC709.

MTC 733

Constructed like MTC723, but MTC1 was mated with MTC716.

MTC735

Constructed like MTC723, but MTC1 was mated with MTC718.

MTC737

Constructed like MTC723, but MTC1 was mated with MTC721.

MTC745

Constructed like MTC723, but MTC390 was mated with MTC708.

MTC747

Constructed like MTC723, but MTC390 was mated with MTC709.

MTC755

Constructed like MTC723, but MTC390 was mated with MTC716.

MTC757

Constructed like MTC723, but MTC390 was mated with MTC718.

MTC759

Constructed like MTC723, but MTC390 was mated with MTC721.

MTC772

Constructed like MTC723, but MTC1 was mated with MTC764.

MTC774

Constructed like MTC723, but MTC390 was mated with MTC764.

MTC789

Constructed like MTC723, but MTC626 was mated with MTC708.

MTC790

Constructed like MTC723, but MTC626 was mated with MTC709.

MTC794

Constructed like MTC723, but MTC626 was mated with MTC716.

MTC795

Constructed like MTC723, but MTC626 was mated with MTC718.

MTC797

Constructed like MTC723, but MTC626 was mated with MTC764.

MTC838

Constructed like MTC390, but MTC390 was mated with MTC808.

MTC842

Constructed like MTC390, but MTC390 was mated with MTC809.

MTC949

Constructed like MTC390, but MTC628 was mated with MTC413.

***E. coli* strains**

MTC257

Plasmid mini-CTX-1-aac was electroporated into SM10 (MTC27), and transformants were selected on LB plates containing 25 μg/mL tetracycline.

MTC296, 410, 413, 417, 467, 488, 808, 809

The appropriate pEXG2-derived knockout plasmids (listed in Table S2) were electroporated into SM10 (MTC27), and transformants were selected on LB plates containing 20 μg/mL gentamycin.

MTC708, 709, 716, 718, 721, 764

The appropriate mini-CTX-1-lux derivatives were electroporated into SM10 (MTC27), and transformants were selected on LB plates containing 25 μg/mL tetracycline.

Modes of plasmid construction

All plasmids constructed in this study were assembled from purified PCR products and restriction enzyme-cleaved plasmid backbones using isothermal assembly [5].

pCTX-1-aacC1

The *aac* gene, encoding gentamycin resistance, was PCR-amplified from plasmid pPSV35 [4] using primers 120 and 121. The resulting fragment was assembled into EcoRI/HindIII-cleaved mini-CTX-1.

pCTX-1-P_{hcnA}-lux

The *hcnA* promoter was PCR-amplified from PA14 genomic DNA using primers 358 and 359 and assembled into EcoRI/BamHI-cleaved mini-CTX-1-lux.

pCTX-1-P_{lasB}-lux

The *lasB* promoter was PCR-amplified from PA14 genomic DNA using primers 82 and 357 and assembled into EcoRI/BamHI-cleaved mini-CTX-1-lux.

pCTX-1-P_{phzA1}-lux

The *phzA1* promoter was PCR-amplified from PA14 genomic DNA using primers 86 and 353 and assembled into EcoRI/BamHI-cleaved mini-CTX-1-lux.

pCTX-1-P_{rhlA}-lux

The *rhlA* promoter was PCR-amplified from PA14 genomic DNA using primers 94 and 356 and assembled into EcoRI/BamHI-cleaved mini-CTX-1-lux.

pCTX-1-P_{rsaL}-lux

The *rsaL* promoter was PCR-amplified from PA14 genomic DNA using primers 360 and 361 and assembled into EcoRI/BamHI-cleaved mini-CTX-1-lux.

pEXG2-ΔambB

The upstream and downstream flanking sequences of the first ~1.5 kb of the *ambB* gene were amplified from PA14 chromosomal DNA using primer pairs 370/371 and 372/373, respectively. A fragment containing the *ambB* gene deletion was generated by stitch PCR using the initial fragments as self-priming templates with primers 371 and 373. The resulting deletion fragment was assembled into EcoRI/HindIII-cleaved pEXG2.

pEXG2-ΔlasR

The upstream and downstream flanking sequences of the *lasR* gene were amplified from PA14 chromosomal DNA using primer pairs 128/129 and 131/132, respectively. A fragment containing the *lasR* gene deletion was generated by stitch PCR using the initial fragments as self-priming templates with primers 129 and 132. The resulting deletion fragment was assembled into EcoRI/HindIII-cleaved pEXG2.

pEXG2-ΔrhlI

The upstream and downstream flanking sequences of the *rhlI* gene were amplified from PA14 chromosomal DNA using primer pairs 182/183 and 184/185, respectively. A fragment containing the *rhlI* gene deletion was generated by stitch PCR using the initial fragments as self-priming templates with primers 183 and 185. The resulting deletion fragment was assembled into EcoRI/HindIII-cleaved pEXG2.

pEXG2-ΔrhlR

The upstream and downstream flanking sequences of the *rhlR* gene were amplified from PA14 chromosomal DNA using primer pairs 178/179 and 180/181, respectively. A fragment containing the *rhlR* gene deletion was generated by stitch PCR using the initial fragments as self-priming templates with primers 179 and 181. The resulting deletion fragment was assembled into EcoRI/HindIII-cleaved pEXG2.

pEXG2-ΔrsaL

The upstream and downstream flanking sequences of the *rsaL* gene were amplified from PA14 chromosomal DNA using primer pairs 226/136 and 225/134, respectively. A fragment containing the *rsaL* gene deletion was generated by stitch PCR using the initial fragments as self-priming templates with primers 134 and 136. The resulting deletion fragment was assembled into EcoRI/HindIII-cleaved pEXG2.

pEXG2-ΔrsaLlasI

The upstream and downstream flanking sequences of the genomic region containing the *rsaL* and *lasI* genes were amplified from PA14 chromosomal DNA using primer pairs 232/173 and 214/227, respectively. A fragment containing the *rsaL-lasI* deletion was generated by stitch PCR using the initial fragments as self-priming templates with primers 214 and 173. The resulting deletion fragment was assembled into EcoRI/HindIII-cleaved pEXG2.

pEXG2-ΔphoB

The upstream and downstream flanking sequences of the *phoB* gene were amplified from PA14 chromosomal DNA using primer pairs 376/377 and 378/379, respectively. A fragment containing the *phoB* gene deletion was generated by stitch PCR using the initial fragments as self-priming templates with primers 377 and 379. The resulting deletion fragment was assembled into EcoRI/HindIII-cleaved pEXG2.

pEXG2-ΔpqsA

The upstream and downstream flanking sequences of the *pqsA* gene were amplified from PA14 chromosomal DNA using primer pairs 186/187 and 188/189, respectively. A fragment containing the *pqsA* gene deletion was generated by stitch PCR using the initial fragments as self-priming templates with primers 187 and 189. The resulting deletion fragment was assembled into EcoRI/HindIII-cleaved pEXG2.

References

1. Simon R, Prieffer, U., and Puhler, A. (1983) A broad host range mobilization system for in vivo genetic engineering: transposon mutagenesis in gram-negative bacteria. Biotechnology (NY) 1: 784-790.
2. Hoang TT, Kutchma AJ, Becher A, Schweizer HP (2000) Integration-proficient plasmids for *Pseudomonas aeruginosa*: site-specific integration and use for engineering of reporter and expression strains. Plasmid 43: 59-72.
3. Becher A, Schweizer HP (2000) Integration-proficient *Pseudomonas aeruginosa* vectors for isolation of single-copy chromosomal *lacZ* and *lux* gene fusions. Biotechniques 29: 948-950, 952.
4. Rietsch A, Vallet-Gely I, Dove SL, Mekalanos JJ (2005) ExsE, a secreted regulator of type III secretion genes in *Pseudomonas aeruginosa*. Proc Natl Acad Sci U S A 102: 8006-8011.
5. Gibson DG, Young L, Chuang RY, Venter JC, Hutchison CA, 3rd, et al. (2009) Enzymatic assembly of DNA molecules up to several hundred kilobases. Nat Methods 6: 343-345.
